# *In vivo* characterization of the novel CD44v6-targeting Fab fragment AbD15179 for molecular imaging of squamous cell carcinoma: a dual-isotope study

**DOI:** 10.1186/2191-219X-4-11

**Published:** 2014-03-06

**Authors:** Anna-Karin Haylock, Diana Spiegelberg, Johan Nilvebrant, Karl Sandström, Marika Nestor

**Affiliations:** 1 Unit of Otolaryngology and Head & Neck Surgery, Department of Surgical Sciences, Uppsala University, Akademiska Sjukhuset, Uppsala SE-751 85, Sweden; 2 Unit of Biomedical Radiation Sciences, Department of Radiology, Oncology and Radiation Science, Uppsala University, Uppsala SE-751 85, Sweden; 3 Division of Protein Technology, School of Biotechnology, AlbaNova University Center, Royal Institute of Technology, Stockholm SE-106 91, Sweden

**Keywords:** Radio-immunodiagnostics, Antibody fragment, CD44v6, Molecular imaging, Fab, ^125^I, ^111^In

## Abstract

**Background:**

Patients with squamous cell carcinoma in the head and neck region (HNSCC) offer a diagnostic challenge due to difficulties to detect small tumours and metastases. Imaging methods available are not sufficient, and radio-immunodiagnostics could increase specificity and sensitivity of diagnostics. The objective of this study was to evaluate, for the first time, the *in vivo* properties of the radiolabelled CD44v6-targeting fragment AbD15179 and to assess its utility as a targeting agent for radio-immunodiagnostics of CD44v6-expressing tumours.

**Methods:**

The fully human CD44v6-targeting Fab fragment AbD15179 was labelled with ^111^In or ^125^I, as models for radionuclides suitable for imaging with SPECT or PET. Species specificity, antigen specificity and internalization properties were first assessed *in vitro. In vivo* specificity and biodistribution were then evaluated in tumour-bearing mice using a dual-tumour and dual-isotope setup.

**Results:**

Both species-specific and antigen-specific binding of the conjugates were demonstrated *in vitro*, with no detectable internalization. The *in vivo* studies demonstrated specific tumour binding and favourable tumour targeting properties for both conjugates, albeit with higher tumour uptake, slower tumour dissociation, higher tumour-to-blood ratio and higher CD44v6 sensitivity for the ^111^In-labelled fragment. In contrast, the ^125^I-Fab demonstrated more favourable tumour-to-organ ratios for liver, spleen and kidneys.

**Conclusions:**

We conclude that AbD15179 efficiently targets CD44v6-expressing squamous cell carcinoma xenografts, and particularly, the ^111^In-Fab displayed high and specific tumour uptake. CD44v6 emerges as a suitable target for radio-immunodiagnostics, and a fully human antibody fragment such as AbD15179 can enable further clinical imaging studies.

## Background

Squamous cell carcinoma of the head and neck region (HNSCC) makes up approximately 5% of all solid cancers in Europe and North America. In 2008, the worldwide incidence of oral cavity cancer was estimated to 263,900 cases [1]. Squamous cell carcinoma (SCC) is the most common histopathological type in head and neck cancer and occurs in the oral cavity, pharynx, sino-nasal cavity and larynx. The oral cavity is presently the predominant site, although pharyngeal cancer is becoming more common due to infections with human papilloma viruses [2,3]. Still, tobacco use is the outstanding risk factor and particularly in combination with alcohol [4,5].

Already at diagnosis, 60% of HNSCC patients have metastatic disease [6], most commonly locoregional metastasis in the lymph nodes in the neck. Treatment of HNSCC generally involves several modalities in combination such as surgery and radiation and, in some cases, chemotherapy and targeted therapy using antibodies. Despite the fact that treatments have improved, there is still a high risk of disease recurrence. In the last 30 years, survival rates have only improved slightly, with the exception of HPV-induced carcinomas [7].

A cornerstone in the work-up of patients with HNSCC is accurate imaging of the primary tumour and metastases, since tumour stage strongly correlates to prognosis and hence the chosen treatment. The imaging methods available in clinical use today are computed tomography (CT), magnetic resonance imaging (MRI), ultrasound and fludeoxyglucose-positron emission tomography/CT (FDG-PET/CT). FDG-PET/CT is a valuable method when searching for metastases and primary tumours. However, its specificity is limited due to increased FDG uptake in benign lymphadenitis, other inflammatory processes or after treatment. Also, small clusters of cancer cells can escape diagnosis due to the limiting size factor of the PET/CT technique [8]. More specific imaging techniques for HNSCC could both improve the delineation of the primary tumour for more precise surgical removal and more accurately find lymph node metastases.

One promising method that may improve the management of patients with head and neck malignancies is radio-immunodiagnostics, which combines the high sensitivity and resolution of a PET or single-photon emission tomography (SPECT) camera with the tumour specificity of an antibody or antibody fragment. Such radiolabelled monoclonal antibodies (mAbs) can be utilized not only in the detection of metastatic lesions and primary tumours but also in applications that include monitoring of tumour response to therapy, dosimetric calculations, and therapy [9]. Although intact mAbs are still most commonly used, they are not considered optimal for radio-immunodiagnostics. Their relatively large size (approximately 150 kDa) tends to cause unfavourable imaging kinetics due to slow plasma clearance, poor tumour penetration and higher risk of eliciting a host antibody response [10-12]. One solution to these obstacles has been to reduce the size of intact antibodies to smaller fragments, achieved either through enzymatic cleavage or by genetic engineering. For instance, a Fab fragment is an antibody structure of about 50 kDa that still binds to antigens but is monovalent with no Fc portion, whereas Fab_2_ fragment antibodies have two antigen-binding Fab portions linked together by disulphide bonds and therefore are divalent with a molecular weight of about 110 kDa. Fragments of the intact antibody may exhibit shorter half-life, faster blood clearance and better tumour penetration, resulting in faster and higher signal-to-noise ratio. Furthermore, the removal of the Fc segment could reduce the non-specific distribution *in vivo* of the mAb via Fc receptors found on normal cells [13]. However, reduction in size can also reduce antibody avidity [14], and the shortened serum half-life, likely due to kidney clearance and lack of Fc-mediated neonatal receptor recycling, may decrease the overall tumour uptake of these small molecules [15].

Receptors on the surface of cells can serve as targets for antibodies and antibody fragments, and if they are expressed specifically by tumour cells, they are excellent targets for radio-immunodiagnostics. There are several promising receptors for radio-immunodiagnostics such as EGFR and isoforms of CD44. CD44 belongs to a family of glycoproteins serving as surface receptors for extracellular matrix components, mainly hyaluronic acid. The receptors are involved in migration and adhesion of cells. Twenty exons encode CD44, and exons 6 to 15, namely variable exons 1 to 10 (v1 to v10), can be alternatively spliced with diverse end products [16]. Most tissues, both epithelial and non-epithelial, express variants of CD44 with the exception of splice variants v4, v6 and v9 which are more sparsely occurring [17]. For CD44v6, the expression in normal tissue is restricted to squamous and transitional epithelium [17,18]. The overexpression of certain CD44 splice variants has been found to be involved in cancer progression, and CD44v6 in particular has been suggested to play a role in tumour formation, invasion, and metastasis formation [16,19]. One proposed mechanism for the increased metastatic potential is binding to extracellular matrix components, enabling invasion and angiogenesis [19,20]. Previous studies have shown overexpression of CD44v6 in squamous cell carcinomas, for example, in the head and neck, lung, skin, oesophagus, cervix and papillary thyroid cancers, and several studies have demonstrated overexpression of CD44v6 in over 90% of primary and metastatic HNSCC [19,21]. This makes CD44v6 a promising candidate marker for targeting of squamous cell carcinoma [22]. A chimeric monoclonal antibody, cMAb U36, targeted at CD44v6 has previously been evaluated both for diagnostic and therapeutic uses with promising results [23-25], as well as with a fully humanized version, BIWA-4, binding to an overlapping epitope in the v6 domain [26,27]. In a previous study, chimeric Fab and Fab_2_ fragments of U36 radiolabelled with ^125^I were characterized *in vitro* and *in vivo* and compared to the intact antibody. Tumour-to-blood ratios and tumour penetration were increased for Fab and Fab_2_ compared with the intact antibody [12].

To date, few antibody fragments toward CD44v6 have been reported, and none of them are fully human with a thoroughly characterized binding site. Thus, to facilitate improved targeting of CD44v6, we have selected *in vitro* characterized fully human Fab fragments, derived from the HuCAL PLATINUM library, which specifically recognize v6-containing isoforms of CD44 [28]. Clones derived from such recombinant antibody repertoires provide a renewable source of human antibodies or antibody fragments that can be expressed in *Escherichia coli*. Furthermore, the recombinant origin of the antibody fragments enables future reformatting to provide molecules that are optimized for either diagnostic or therapeutic applications [29]. One main candidate, AbD15179, demonstrated optimal affinity and slow dissociation from the tumour cells and was chosen for further evaluation. To our knowledge, this study generated the first and most thoroughly characterized fully human Fab fragment specific for CD44v6 [28].

The aim of this study was primarily to assess the *in vivo* tumour targeting capabilities of the novel, fully human, CD44v6-targeting antibody fragment AbD15179. The Fab fragment was first evaluated for species specificity using surface plasmon resonance (SPR) and was then labelled with ^111^In or ^125^I, as models for radionuclides suitable for imaging with SPECT or PET. Specific binding and internalization of labelled conjugates was evaluated in CD44v6-expressing SCC cells *in vitro*, and *in vivo* binding specificity and biodistribution studies were then performed using ^111^In- or ^125^I-labelled Fab fragments in a dual-isotope study in tumour-bearing mice with xenografts of varying CD44v6 expression.

## Methods

### Antibody fragment AbD15179

The CD44v6-binding Fab fragment AbD15179 was supplied from AbD Serotec (Kidlington, UK). It was selected from an array of 13 different human antibody fragments, all recognizing CD44v6. The selection and production of this antibody fragment have been described previously [28]. The native Fab fragment is referred to as AbD15179 throughout this paper. AbD15179 was supplied in 3× PBS (0.72 μg/ml) and stored at −80°C. The fragment used for ^111^In-labelling was separated by size-exclusion chromatography on a NAP-5 column (Amersham Biosciences, Uppsala, Sweden) pre-equilibrated with purified (MilliQ, Millipore Corp., Billerica, MA, USA) water. It was then freeze-dried and stored at −20°C before use in order to facilitate buffer exchange and concentration adjustments.

### Biosensor evaluation of species specificity of AbD15179

Two 16-mer peptides corresponding to the human (CGNRWHEGYRQTPREDS) and mouse (CQNGWQGKNPPTPSEDS) forms of the CD44v6 antigen used to generate AbD15179 were produced by peptide synthesis (Elim Biopharmaceuticals, Hayward, CA, USA). The peptide antigens were individually conjugated to bovine serum albumin (BSA) through N-terminal cysteine residues. Purified recombinant CD44v3-10 (see [28] for details) and the two carrier-conjugated peptides were immobilized on a ProteOn XPR36 general layer medium biosensor chip (Bio-Rad, Hercules, CA, USA) using amine coupling. This yielded immobilization levels of approximately 1,000 (CD44 isoform) and 300 (BSA-peptides) response units, respectively. A CD44 isoform that lacks the v6 region (CD44v3-10Δv6) was included as a negative control in one channel. Dilution series of AbD15179 (0.6 to 50 nM) were injected at 25°C with a flow rate of 50 μl/min. PBS containing 0.05% Tween 20 (pH 7.4) was used as running buffer, and the surfaces were regenerated using 10 mM HCl. The chimeric anti-CD44v6 monoclonal antibody U36 [30] was used as a positive control. Binding curves were normalized using a blank ligand channel and a parallel buffer injection prior to fitting to a 1:1 Langmuir isotherm using the ProteOn manager software version 3.1.0.6 (Bio-Rad).

### Cell lines

The human HNSCC cell line H314 (obtained from European Collection of Cell Cultures, Salisbury, UK), derived from floor of mouth, was cultured in a one-to-one mixture of Ham's F12 and Dulbecco's modified eagle medium (DMEM), supplemented with 10% foetal calf serum, 2 mM l-glutamine, and antibiotics (100 IU penicillin and 100 μg/ml streptomycin). H314 has demonstrated an average antigen density of approximately 650,000 CD44v6 receptors [31]. The SCC cell line A431 (obtained from the American Type Culture Collection, Manassas, VA, USA), derived from vulva, was cultured in Ham's F10, with the same supplements. A431 has been shown to be a highly CD44v6-expressing cell line with approximately 3,000,000 receptors per cell [32]. As a negative control, the breast cancer cell line MDA-MB-231 (obtained from the American Type Culture Collection) was used. It was cultured in DMEM, with the same supplements as above. This cell line has demonstrated no detectable CD44v6 expression [31]. The cells were incubated at 37°C in an atmosphere containing humidified air with 5% CO_2_. The cells used in the monolayer culture experiments were trypsinized and seeded in separate dishes used for experiments 2 to 3 days prior to the studies.

### ^125^I and ^111^In labelling of AbD15179

^125^I- and ^111^In-labelled AbD15179 have previously been extensively evaluated *in vitro*[31]. In the present study, AbD15179 was labelled with ^125^I (PerkinElmer, Waltham, MA, USA) using direct chloramine T labelling [33] (Sigma-Aldrich, St. Louis, MO, USA) as previously described [28,31]. Generally, a stock solution of 10 MBq ^125^I was mixed with 100 μl PBS, and 100 μg of the Fab fragment (140 μl, 0.72 mg/ml in PBS) was added. The reaction was initiated by the addition of chloramine T (Sigma-Aldrich) in water (40 μl, 4 mg/ml) and was quenched after a 5-min incubation on ice by the addition of sodium metabisulphite (Merck, Whitehouse Station, NJ, USA) in water (80 μl, 4 mg/ml). Each labelled protein was separated from non-reacted ^125^I and low-molecular-weight reaction components on a NAP-5 column pre-equilibrated with PBS. ^125^I-labelled AbD15179 is referred to as ^125^I-Fab in this paper.

Labelling of AbD15179 with ^111^In (Mallinckrodt Medical B.V., St. Louis, MO, USA) was done using CHX-A″-DTPA labelling as described previously [31,34]. The chelator CHX-A″-DTPA used for ^111^In labelling exhibits high affinity for several isotopes that are suitable for either diagnostic or therapeutic purposes, including ^111^In, ^86^Y, ^90^Y and ^177^Lu. In short, to 75 μg AbD15179 solution (15 μl, 5 mg/ml in borate buffer, pH 9), 4.1 μl of CHX-A″-DTPA solution (1 mg/ml in borate buffer, 0.07 M, pH 9) was added, corresponding to a chelator-to-AbD15179 molar ratio of 5:1. The reaction mixture was incubated overnight at room temperature, and the conjugated antibody was then separated from free CHX-A″-DTPA using a NAP-5 column equilibrated with acetate buffer (0.2 M, pH 5.5). Approximately 10 MBq of ^111^In in acetate buffer was then added to the conjugated AbD15179, and the reaction mixture was allowed to incubate for 30 min at room temperature. Labelled Fab fragment was then separated from the non-reacted radionuclide and low-molecular-weight reaction components by using a NAP-5 column pre-equilibrated with PBS. ^111^In-labelled AbD15179 is referred to as ^111^In-Fab in this paper.

To determine the purity and stability of the labelled antibody fragments, instant thin-layer chromatography (ITLC) was performed on ^125^I- and ^111^In-labelled conjugates. The purity of the samples taken immediately, as well as stored at 4°C for 48 h, was analysed. Approximately 1 μl of the conjugate was placed on a chromatography strip (Biodex, Shirley, NY, USA) and placed into a ‘running buffer’ (70% acetone or 0.2 M citric acid for ^125^I and ^111^In, respectively), followed by measurements on a Cyclone Storage Phosphor System (PerkinElmer). The data were analysed using the OptiQuant image analysis software (PerkinElmer). Furthermore, stability in murine serum was evaluated using sodium dodecyl sulphate polyacrylamide gel electrophoresis (SDS-PAGE) analysis (200 V constant) on NuPAGE® 4-12% Bis-Tris gel (Novex, Thermo Fisher Scientific, Waltham, MA, USA). Briefly, 1.8 μg of ^125^I-Fab or ^111^In-Fab was incubated in 120 μl of murine serum (42% in PBS, pH 7.4) for 60 min at 37°C. After incubation, the samples were treated with NuPAGE® LDS Sample Buffer according to the manufacturer's instructions and loaded on the gel. One well was loaded with 4 μl of HiMark™ Pre-Stained Protein Standard (Novex®). Measurements were performed on a Cyclone Storage Phosphor System, and the data were analysed using the OptiQuant image analysis software.

### *In vitro* binding, specificity and internalization

*In vitro* binding and specificity were assessed for ^125^I-Fab, and binding, specificity and internalization were assessed for ^111^In-Fab using both H314 and A431 cells. For binding and specificity measurements, 10 nM ^125^I-Fab or ^111^In-Fab was added to each dish and incubated for 2 to 24 h. To some of the dishes, a 100-time molar excess of unlabelled antibody fragment was simultaneously added for assessment of binding specificity. After incubation, the cells were washed with serum-free medium to remove unbound antibody fragment, detached with 0.5 ml trypsin at 37°C and resuspended in 1 ml complete medium. A sample of 0.5 ml of the cell solution was taken for cell counting, and the remaining 1 ml was collected for radioactivity measurements.

For internalization measurements, 10 nM ^111^In-Fab was added to each dish and incubated for 2 to 24 h using the following conditions: (*i*) incubation at 37°C to allow internalization of the conjugate, (*ii*) incubation at 37°C in the presence of a 100-time molar excess of unlabelled antibody for assessment of binding specificity and (*iii*) incubation at 0°C for correction of acid-resistant surface binding at non-internalizing conditions. After incubation, the cells were washed with serum-free medium to remove unbound antibody and incubated at 0°C with 20 mM HCl/150 mM NaCl (pH 1.7) for 15 min to release antibodies bound to the cell surface. The supernatant was collected, and the radioactivity was counted in a gamma counter to assess the amount of membrane-bound conjugates. Finally, the cells were lysed with 0.1 M NaOH/1% Triton X-100, and the radioactivity was measured in a gamma counter to assess the amount of internalized conjugates. After correction for acid-resistant surface binding, the internalization at 37°C was calculated as the percentage of the total amount of radioactivity specifically bound (internalized and surface-bound) to the cells.

### *In vivo* specificity

Tumour xenografts were formed by subcutaneous inoculation of approximately 8 × 10^6^ A431 cells (high CD44v6 expression) suspended in 300 μl injected medium in the right posterior leg, and approximately 8 × 10^6^ MDA-MB-231 cells (no CD44v6 expression) in the left posterior leg, in five nude balb/c (nu/nu) female mice. The mice were housed in a controlled environment and fed *ad libitum*. All experiments complied with the current Swedish law and were performed with permission granted by the Uppsala Committee of Animal Research Ethics.

After allowing the tumours to grow for 2 weeks, the animals were injected with^125^I-Fab (5 μg in 200 μl PBS, 100 kBq, rendering a specific activity of 20 kBq/μg) in the tail vein. After 24 h, the animals were euthanized with a mixture of ketamine and xylazine followed by heart puncture, and blood was aspirated and weighed. Tumours were excised and weighed. Radioactivity content of the blood and tumours was measured in a gamma counter, together with three injection standards of 200 μl ^125^I-Fab (volume of injection). A mean injected dose was calculated from the injection standards, and the injected activity of ^125^I-Fab for each mouse was calculated by subtracting the residual activity in the syringe from the mean injected dose. Radioactivity uptake in the organ was calculated as percent of injected activity per gram of tissue (%ID/g). Tumour-to-organ ratio was calculated as activity/g_tumour_ divided by activity/g_organ_.

### Biodistribution

Tumour xenografts of A431 and H314 cells (high and moderate CD44v6 expression, respectively) were formed in 20 nude balb/c (nu/nu) female mice as described above. The mice were housed in a controlled environment and fed *ad libitum*. The experiments complied with the current Swedish law and were performed with permission granted by the Uppsala Committee of Animal Research Ethics.

Three weeks after tumour cell injection, the animals received an intravenous injection via the tail vein with^125^I-Fab (100 kBq, specific activity of 20 kBq/μg) and ^111^In-Fab (100 kBq, specific activity of 20 kBq/μg), totally 5 μg Fab fragments in 200 μl PBS. At 6 h (*N* = 5), 24 h (*N* = 5), 48 h (*N* = 5) and 72 h (*N* = 5) post injection, the animals were euthanized with a mixture of ketamine and xylazine followed by heart puncture, and blood was aspirated and weighed. The tumours, salivary glands, thyroid (*en bloc* with larynx), tongue, liver, kidneys, spleen, urinary bladder, colon, upper gastrointestinal tract, skin, bone and muscle were excised and weighed, and radioactivity was measured in a gamma counter. The tail and the rest of the body were also separated, weighed and measured in a gamma counter, together with three injection standards for each group. The injected dose was calculated as described above. Radioactivity uptake in the organ was calculated as %ID/g. Uptake was calculated as percent of injected activity per organ. Tumour-to-organ ratio was calculated as activity/g_tumour_ divided by activity/g_organ_.

### Statistical analyses

Statistical analyses were performed using GraphPad Prism Version 5.02 for Windows (GraphPad Software, Tumour Biol Inc., La Jolla, CA, USA, http://www.graphpad.com). For *in vivo* specificity studies, the differences in ^125^I-Fab uptake between MDA-MB-231 tumours and A431 tumours were assessed using a two-tailed paired *t* test and were considered statistically significant if *P* < 0.05. In the biodistribution studies, the data are presented as the mean ± standard deviation (SD). The significance of differences between the groups over time was tested with one-way analysis of variance (ANOVA) with Newman-Keuls multiple comparison test. The differences were considered statistically significant if *P* < 0.05. The differences in ^125^I-Fab or ^111^In-Fab uptake between H314 tumours and A431 tumours were assessed using repeated measures ANOVA with Newman-Keuls multiple comparison test and were considered statistically significant if *P* < 0.05.

## Results

### Biosensor evaluation of species specificity of AbD15179

**Figure 1:**
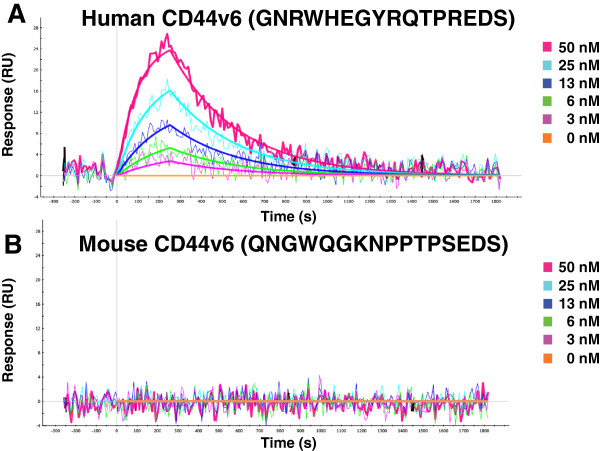
**SPR evaluation of the Fab fragment AbD15179 to (A) human and (B) mouse CD44v6 peptides.** AbD15179 clearly bound to the human CD44v6 peptide, whereas no signal was detected for mouse CD44v6.

The Fab fragment AbD15179 bound strongly to the human v6 peptide, whereas no binding was detected for the murine form of the target (Figure 1). The same binding pattern was observed for U36 as expected. The low nanomolar target binding affinity (*K*_D_ < 7 nM) of AbD15179 reported previously [28] was verified using triplicate injections and two different immobilization levels of CD44v6-10. As observed earlier, the affinity for the conjugated peptide target was roughly tenfold lower than that for the full-length extracellular domain CD44v3-10, which was a result of both a faster dissociation rate constant and a slower association.

### Labelling

Labelling yields for ^125^I and ^111^In labelling of AbD15179 for *in vitro* studies were 50% and 39%, rendering specific activities of 50 and 52 kBq/μg, respectively. The labelling yield for ^125^I labelling of AbD15179 for *in vivo* specificity studies was 66%, and for biodistribution studies 74%, rendering specific activities of 66 and 74 kBq/μg, respectively. Specific activities were adjusted to 20 kBq/μg for all *in vivo* conjugates, using unlabelled AbD15179, before injections. The purity of the labelled proteins was >99.9% according to the ITLC analysis. The labelling yield for ^111^In labelling of AbD15179 for biodistribution studies was 63%, resulting in a specific activity of 84 kBq/μg, and the purity of the labelled protein was >96% according to the ITLC analysis. The purity of labelled conjugates stored in PBS at 4°C was re-analysed 48 h post injection (p.i.) and was unchanged according to the ITLC analysis. SDS-PAGE analysis of the sample incubated in serum for 1 h at 37°C showed a single radioactivity peak corresponding to the size of the intact Fab fragment for both ^125^I-Fab and ^111^In-Fab, indicating high stability of the fragments.

### *In vitro* binding, specificity and internalization

**Figure 2:**
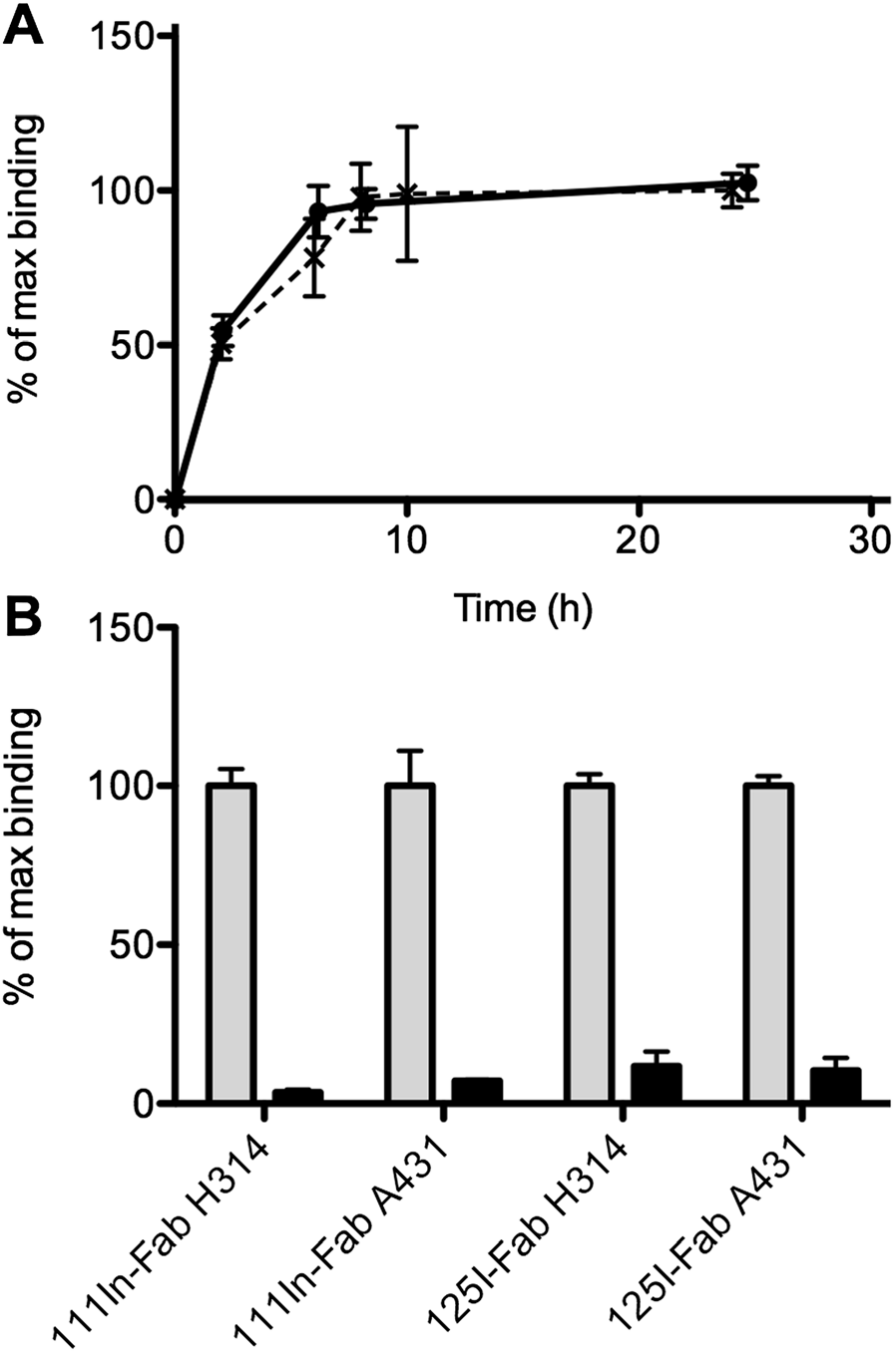
**Cellular uptake (A) and specificity (B) of **^**111**^**In-Fab and **^**125**^**I-Fab in cultured cells. (A)** Cellular uptake of ^111^In-Fab (straight line) and ^125^I-Fab (broken line) during incubation for 0 to 24 h in H314 cells. Error bars represent SD (*N* = 3 to 6). **(B)** Specificity of ^111^In-Fab and ^125^I-Fab in H314 and A431 cells. Grey bars show uptake in unblocked cells, whereas black bars show uptake in cells blocked with an excess of unlabelled AbD15179. Error bars represent SD (*N* = 3 to 8).

Cellular binding of ^111^In-Fab and ^125^In-Fab increased with time, levelling out at approximately 6 h in both cell lines. Furthermore, binding of ^111^In-Fab and ^125^In-Fab was blocked by an excess of unlabelled AbD15179 in both cell lines, demonstrating specific binding of the conjugates (Figure 2). Internalization studies of ^111^In-Fab demonstrated no detectable internalization after 24 h in neither A431 nor H314 cells. Furthermore, ^125^I- and ^111^In-labelled AbD15179 have previously been extensively evaluated *in vitro*[31]. In brief, both ^125^I-Fab and ^111^In-Fab bound specifically to CD44v6-expressing cells and demonstrated a general biphasic appearance of one high- and one low-affinity binding event. The population of high-affinity binders was significantly higher for ^111^In-Fab than that for ^125^I-Fab, which resulted in longer retention of ^111^In-Fab compared to ^125^I-Fab on SCC cells. The equilibrium dissociation constant (*K*_D_) for the high-affinity population (measured using cell-based real-time measurements and interaction analysis) was approximately 8 ± 2 nM and did not significantly differ between ^125^I- or ^111^In-labelled AbD15179 on the H314 or A431 cells [31].

### *In vivo* specificity

The specificity of AbD15179 was assessed *in vivo* by comparing the uptake of ^125^I-Fab in CD44v6-negative (MDA-MB-231) and CD44v6-positive (A431) tumours 24 h p.i. in tumour-bearing mice. At the time of the experiments, median tumour weight for MDA-MB-231 tumours was 131 mg, generally ranging between 90 mg (lower quartile) and 338 mg (upper quartile). For the A431 tumours, the median weight was 62 mg, generally ranging between 43 and 95 mg. There was no correlation between the tumour weight and %ID/g of ^125^I-Fab. Tumour uptake differed significantly between the CD44v6-negative and CD44v6-positive tumours, with the CD44v6-positive A431 tumours displaying more than two times higher uptake than the CD44v6-negative MDA-MB-231 tumours (0.66 ± 0.13 (%ID/g ± SD) and 1.34 ± 0.26 %ID/g for MDA-MB-231 and A431 tumours, respectively).

### Biodistribution

**Figure 3:**
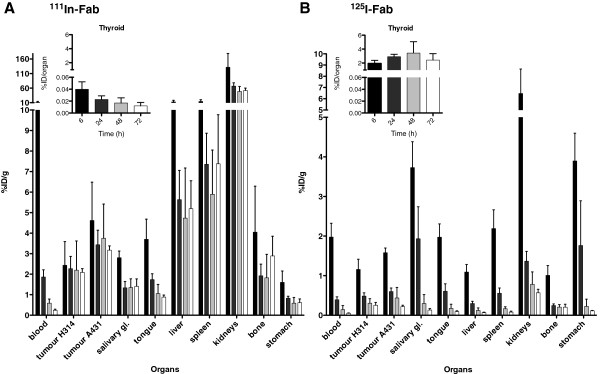
**Biodistribution of (A) **^**111**^**In-Fab and (B) **^**125**^**I-Fab in tumours and organs of SCC tumour-bearing mice.** The target antigen CD44v6 is expressed moderately in H314 tumours and to a high extent in A431 tumours. The animals were sacrificed at 6-h (black bars), 24-h (dark grey), 48-h (light grey) and 72-h (white) post injection. The data are expressed as percentages of injected activity per gram of tissue, %ID/g, except for the thyroid (inset), which is expressed as percentages of injected activity per organ. Error bars represent SD (*N* = 5)

The biodistribution of AbD15179 was assessed *in vivo* by comparing the uptake of ^125^I-Fab and ^111^In-Fab in mice bearing both CD44v6-moderate (H314) and CD44v6-positive (A431) tumours. At the time of the experiments, the median tumour weight for the H314 tumours was 23 mg, generally ranging between 17 mg (lower quartile) and 33 mg (upper quartile), and the median tumour weight for the A431 tumours was 105 mg, generally ranging between 86 and 294 mg. There was no correlation between tumour weight and %ID/g of ^125^I-Fab or ^111^In-Fab. The biodistribution data of the selected organs of the ^125^I-Fab and ^111^In-Fab fragments in tumour-bearing mice are shown in Figure 3. The selected organs were the ones in the vicinity of the head and neck region, tongue, salivary glands and thyroid, as well as the organs of high interest for radio-immunotargeting such as liver, kidneys and spleen. Generally, ^111^In-Fab displayed not only higher uptake of radioactivity and slower clearance in most organs compared with ^125^I-Fab but also a higher tumour-to-blood ratio and a clearer discrimination between high- and moderate-CD44v6-expressing tumours. The biodistribution data are described in more detail below.

For ^111^In-Fab, the activity in tumours remained relatively stable over time in both the moderate (H314) and high (A431) CD44v6-expressing tumour models, with no significant changes in tumour uptake over time (Figure 3A). ^111^In-Fab displayed a clear difference in tumour uptake between the moderate- and high-CD44v6-expressing tumours, ranging between 1.5 to 2 times higher for the high-CD44v6-expressing A431 tumours (calculated for each individual mouse) throughout the study. At 6 h p.i., the percentage of the injected dose per gram of ^111^In-Fab in the moderate-CD44v6-expressing H314 tumours was 2.4 ± 1.1 (SD) and remained at this level. The uptake in the high-CD44v6-expressing A431 tumours was significantly higher, being 4.6 ± 1.9 %ID/g at 6 h p.i., with a slight, although not significant, decrease over time to 3.2 ± 0.2 %ID/g at 72 h p.i. For blood, bone and organs, ^111^In-Fab demonstrated highest activities at the first time point (6 h), declining with time. The kidneys displayed the highest activity (131 ± 47 %ID/g at 6 h), which rapidly decreased to 67 ± 11 %ID/g by 24 h. The second highest normal organ uptake was observed in the liver (13 ± 5.6 %ID/g at 6 h), rapidly decreasing to 5.6 ± 1.4 %ID/g by 24 h.

^125^I-Fab generally displayed lower uptake values than ^111^In-Fab in both tumours and normal organs, with a more pronounced clearance and decrease in activity over time (Figure 3B). At 6, 24, and 48 h p.i., ^125^I-Fab displayed a difference in tumour uptake between the high- and moderate-CD44v6-expressing tumours, although not as pronounced as for ^111^In-Fab, ranging between 1.3 and 1.4 times higher for the high-CD44v6-expressing A431 tumours compared to the medium-CD44v6-expressing H314 tumours. At 72 h p.i., there was no difference in activity between the tumours. A clear decrease in tumour activity was seen from 6 to 24 h p.i. in both tumour models. In high-CD44v6-expressing A431 tumours, this decrease was also significant between 48 and 72 h p.i. At 6 h p.i., the activity of ^125^I-Fab in the moderate-CD44v6-expressing tumours was 1.1 ± 0.6 %ID/g, steadily decreasing over time, reaching 0.25 ± 0.07 %ID/g at 72 h p.i. In the high-CD44v6-expressing A431 tumours, the activity of ^125^I-Fab was 1.6 ± 0.1 %ID/g at 6 h, decreasing to 0.22 ± 0.03 %ID/g at 72 h p.i. As for the ^111^In conjugate, the organ with the highest activity was the kidney (6.5 ± 2.1 %ID/g at 6 h), with the activity rapidly decreasing to 1.4 ± 0.2 at 24 h. The stomach and salivary glands displayed high initial values, both rapidly decreasing to below 0.3 %ID/g 48 h p.i. The exception to this trend was uptake in the thyroid, where no decrease in activity was observed (Figure 3B, inset).

**Figure 4:**
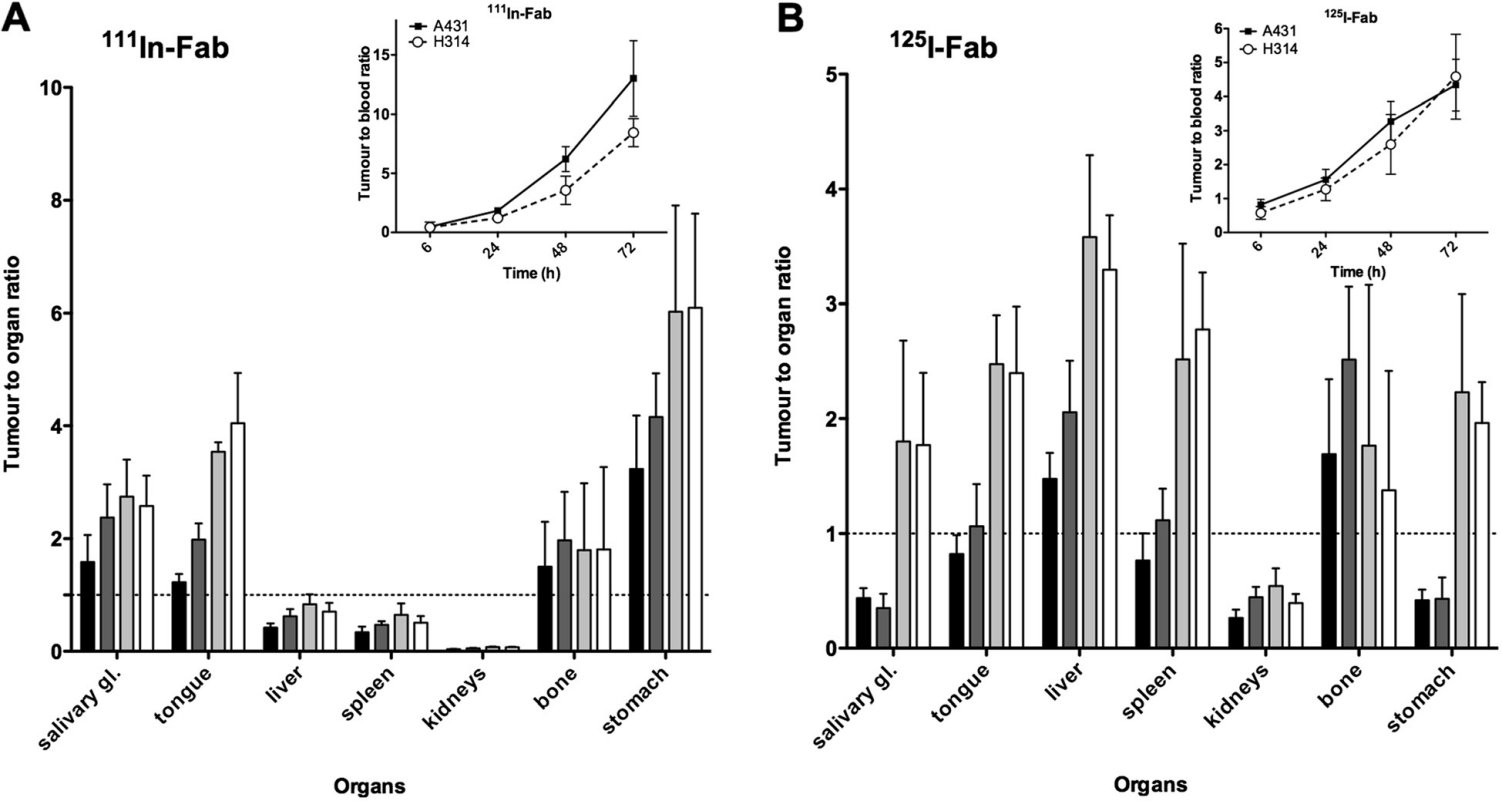
**Tumour-to-organ ratios of (A) **^**111**^**In-Fab and (B) **^**125**^**I-Fab in tumours and organs of SCC tumour-bearing mice.** A431 tumours were used as reference for tumour-to-organ ratio calculations. The animals were sacrificed at 6 h (black bars), 24 h (dark grey), 48 h (light grey) and 72 h (white) post injection. Inset: tumour-to-blood ratios of radiolabelled Fab for both A431 and H314 tumours. Error bars represent SD (*N* = 5).

In order to assess the selectivity of tumour targeting for the ^111^In-Fab and ^125^I-Fab conjugates, tumour-to-normal tissue ratios were calculated based on the high-CD44v6-expressing A431 tumours (Figure 4), and tumour-to-blood ratios were calculated for both tumour types (Figure 4, insets). The tumour-to-organ ratios for ^111^In-Fab increased over time for most organs of interest including the blood (Figure 4A) and were highest at 48 and 72 h p.i. The tumour-to-blood ratios for both A431 and H314 tumours increased over time with a ratio of over 10 for A431 tumours at 72 h p.i. (Figure 3A, inset). Also, for ^125^I-Fab, the tumour-to-organ ratios increased over time (Figure 3B). The tumour-to-blood ratios increased with time for both tumours with a maximum ratio at 72 h above 4 in both A431 and H314 tumours (Figure 3B, inset). In the spleen, liver and kidney, higher tumour-to-normal tissue ratios generally were observed with ^125^I-Fab, while the opposite behaviour was seen in the blood, tongue and salivary glands, where ^111^In-Fab displayed higher ratios.

## Discussion

Detecting small metastases and small primary tumours in patients with HNSCC could improve staging and consequently also the treatment of these patients. Tumour stage correlates closely to survival, and if treatment can be individualized through better staging, survival rates should improve. Although there are several diagnostic methods available today, major difficulties in finding small tumours and detecting minimal residual disease still remain. A specific targeting molecule labelled with an appropriate radionuclide utilized in a SPECT or PET setting could add considerably to existing methods. This kind of molecular imaging offers the ability to visualize, characterize and measure processes on molecular and cellular levels non-invasively in living systems. Additionally, fused imaging modalities such as PET/CT, PET/MRI and SPECT/CT have enabled the combination of anatomy and molecular events into a single image [9]. The antibody fragment AbD15179 recognizes the CD44v6 antigen, which has shown consistent expression in many primary squamous cell carcinomas but only in a subset of normal tissues [17]. The potential of CD44v6 as a target for radio-immunotargeting agent has been demonstrated in previous studies using full-length antibodies [23,24]. However, very few antibody fragments toward this target have been reported, and none of them are fully human with a thoroughly characterized binding site. This study represents, to our knowledge, the first *in vivo* evaluation of a fully human CD44v6-targeting antibody fragment.

The objective of this study was to evaluate, for the first time, the *in vivo* properties of the CD44v6-targeting fragment AbD15179, labelled with ^111^In or ^125^I, and to assess its utility as a targeting agent for radio-immunodiagnostics of CD44v6-expressing tumours. Species specificity, antigen specificity and internalization properties were first assessed *in vitro. In vivo* specificity and biodistribution was then evaluated in tumour-bearing mice, using a dual-tumour and dual-isotope setup. An advantage of using this setup is the possibility to evaluate uptake in a low/moderate-CD44v6-expressing tumour vs. a high-CD44v6-expressing tumour, normalizing each dataset to the same mouse. This can minimize the effects of potential differences among groups of experimental animals such as tumour size and injection variability. In the same way, the effects of ^111^In vs. ^125^I labelling of the fragment can be evaluated.

*In vitro*, both species-specific and antigen-specific binding of the conjugates were demonstrated, with no detectable internalization. The lack of binding of AbD15179 to the murine v6 peptide was expected, since 9 of the 16 residues in the peptides differ between the murine and human variants [35]. Furthermore, the two consecutive proline residues, which are not present in the human sequence, may distort the conformation of murine CD44v6 in the targeted region. The low internalization was also in line with previous studies of CD44v6, which have shown that CD44v6 is commonly not an internalizing receptor [32,36]. Generally, high tumour internalization of a radioconjugate is considered beneficial for radiometals such as ^111^In, since internalization of a residualizing radiometal will cause long tumour retention times, often outweighing disadvantages of radiometals such as accumulation in the liver, spleen and kidneys [37]. Thus, the lack of internalization shown *in vitro* could favour a radiohalogen before a radiometal as a radiolabel *in vivo* for AbD15179. However, in a previous *in vitro* study of this fragment, we could demonstrate that the binding characteristics of ^111^In-Fab were more favourable than those for ^125^I-Fab, even in a non-internalizing setting, resulting in longer retention of ^111^In-Fab compared to that of ^125^I-Fab on SCC cells [31]. Thus, we considered ^111^In-Fab to be an interesting candidate for the subsequent *in vivo* studies, despite an expected low internalization rate.

*In vivo*, we first demonstrated a CD44v6-dependent tumour uptake for AbD15179, with a markedly higher tumour uptake of ^125^I-Fab in high-CD44v6-expressing tumours compared to the CD44v6-negative tumours, establishing the specificity of the conjugate also *in vivo*. Tumour uptake (%ID/g) differed significantly between the CD44v6-negative and CD44v6-positive tumours, with the CD44v6-positive A431 tumours displaying more than two times higher uptake than the CD44v6-negative MDA-MB-231 tumours. This is in line with the values obtained at the same time point (24 h p.i.) in the subsequent biodistribution study in this paper, in which high-CD44v6-expressing A431 tumour uptake of ^125^I-Fab was approximately 1.3 times higher than the uptake in the moderate-CD44v6-expressing H314 tumours. However, at later time points, the difference in tumour uptake between moderate- and high-CD44v6-expressing tumours was diminished for ^125^I-Fab, whereas ^111^In-Fab displayed a clear difference in tumour uptake between the moderate- and high-CD44v6-expressing tumours, ranging between 1.5 to 2 times higher for the high-CD44v6-expressing tumours throughout the study. Thus, we conclude that ^111^In-Fab displayed higher *in vivo* target sensitivity at the administered dose.

^111^In-Fab also demonstrated a higher tumour uptake than ^125^I-Fab at all time points, being more than 15 times higher at the last time point (72 h p.i.). The accumulation and slower dissociation of ^111^In-Fab in tumours were in line with the slower off-rate of ^111^In-Fab compared to ^125^I-Fab observed on these tumour cells *in vitro* shown in a preceding study [31] using non-internalizing conditions, as discussed previously. However, the effect seen *in vivo* could nevertheless in part be due to some internalization of the ^111^In-Fab by the tumour cells as the cellular membrane renews itself, even though our *in vitro* experiments showed no detectable internalization of ^111^In-Fab into either H314 or A431 tumour cells up to 24 h of incubation at 37°C.

For successful imaging, the contrast between tumour and surrounding tissue is of even higher importance than the total tumour uptake. In the present study, tumour-to-blood ratios were above 1 already at 24 h p.i. for both conjugates and continued to increase throughout the study. This can be compared to previous studies, where successful CD44v6 imaging in tumour-bearing mice using both ^111^In [38] and ^124^I [39] coupled to a CD44v6-targeting antibody was performed, despite three to four times lower tumour-to-blood ratios at the time of imaging than in our current study. We believe that this is very promising and demonstrates that imaging with ^111^In- or ^124^I-labelled AbD15179 is indeed feasible.

In general, ^125^I-labelled Fab fragments were cleared from the circulation more rapidly than the ^111^In-Fab fragments. This is in line with several other studies using ^125^I- and ^111^In-labelled molecules, in which the ^125^I-labelled compounds have generally shown lower uptake and faster clearance from organs. The reason for this has been suggested to be dehalogenation of the radioiodinated protein or exchange of ^111^In into iron-binding proteins [37]. Another explanation for the more rapid clearance could be a higher amount of aggregation of ^125^I-Fab than for ^111^In-Fab [31], resulting in an increase in the effective size and, thus, clearance from the circulation via a size-dependent mechanism, as has been shown previously for liposomes [40].

Furthermore, ^111^In-Fab displayed a considerably higher uptake than ^125^I-Fab in the liver, spleen and kidneys. These observations are also in agreement with those reported in the literature when ^125^I and ^111^In are compared [37]. The observed increase of ^111^In in the liver could be a result of antibody clearance or exchange with iron-binding protein or a mixture of both processes. The accumulation of ^111^In in the kidneys can probably be attributed to the active filtration of the fragments and probably to the subsequent exchange of ^111^In into non-binding proteins within this organ [41]. The property of the liver and kidneys to accumulate ^111^In and clear it more slowly is a distinct disadvantage of ^111^In and may limit its use, although this should be balanced by the fact that for ^111^In-Fab, the magnitude of tumour uptake was several times higher than seen with the radioiodinated fragment. Additionally, several preclinical and clinical experiments have shown that undesirable renal uptake of ^111^In can be reduced with pretreatment with e.g. cationic amino acids such as lysine [42-44]. Moreover, with the aim to discriminate between tumour/metastases and surrounding tissue in the head and neck cancer region, renal uptake is of less concern for imaging of head and neck cancer, although it may limit the amount of activity that can be administered to patients due to dose-limiting toxicity to the kidneys.

For imaging in head and neck cancer, tumour-to-tissue contrast of muscle, salivary glands, thyroid and, to some extent, bone is important, in addition to the tumour-to-blood ratio. Some normal mouse tissues containing squamous and transitional epithelium, such as tongue and skin, express murine CD44v6. However, we demonstrate in this study that AbD15179 does not cross-react with the murine CD44v6. Thus, studies with fragments cross-reactive with murine CD44v6 would be needed to study the effect of CD44v6 expression in normal tissues in a mouse model. Consequently, assuming that only non-CD44v6-mediated uptake was measured, our data indicate that there was no unspecific accumulation of ^125^I-Fab or ^111^In-Fab in any normal tissue. The transferability of the results to targeting HNSCC in humans is still somewhat limited, at least for organs with known CD44v6 expression as the epithelia of the oral cavity [17,18]. However, previous clinical studies of CD44v6-binding antibodies have been performed for several CD44v6-tageting antibody conjugates for both radio-immunotherapy and radio-immunodiagnostics [23,26,45,46], demonstrating no selective accumulation at non-tumour sites, except in faeces and urine [25], and only minimal accumulation of activity in mouth, lung, spleen, kidney, bone marrow and scrotal area [27]. Since we have previously shown that AbD15179 at least partly binds to the same epitope as these antibodies [28], this is promising for the future clinical applicability of our fragment.

The isotopes that have physical and nuclear properties suitable for *in vivo* imaging use are relatively limited. In this study, we used ^111^In as a model for radiometals, and ^125^I as a model for radiohalogens. The choice of radionuclide and the labelling method is an important aspect in successful radio-immunotargeting, and additional improvements in tumour-to-blood ratio could probably be achieved by further optimizing or changing labelling methods or radionuclides. For ^111^In labelling, a CHX-A″-DTPA chelator-to-Fab molar ratio of 5:1 was chosen. It is possible that a different molar ratio could influence targeting properties of the conjugate. However, in a previous study, conjugation of ^111^In was studied at three different molar ratios (5:1, 10:1 and 20:1) of CHX-A″-DTPA chelate to panitumumab Fab_2_*in vitro* and *in vivo*. The different ratios had minimal effect on the immunoreactivity and specific binding [11]. For radioiodination, direct CAT labelling was used, as it is a fast and simple method with high immunoreactive fraction yield, rendering high specific activity. ^125^I was used as a surrogate for radionuclides more suitable for imaging such as ^124^I, or ^131^I for therapy. The thyroid uptake was relatively high for ^125^I-Fab, most likely due to uptake of the free lipophilic iodine catabolites after proteolytic degradation of the antibody fragment. Likewise, the initially high uptake in salivary glands and stomach indicates iodine catabolites, since the Na^+^/I^−^ symporter mediates active I^−^ transport also in these tissues [47]. Values in the same order have previously been reported for directly labelled ^125^I antibody conjugates [39,48]. By using other labelling strategies or by blocking thyroid uptake using, e.g. potassium iodide, thyroid uptake may possibly be reduced [48].

Although intact monoclonal antibodies have been considered candidates for radio-immunodiagnostics due to their specificity, the long residence time in the blood, of days to weeks, limits their applicability. Several studies have demonstrated that the size of antibody-based imaging agents is inversely related to their blood clearance [9]. Consequently, normal tissue exposure will be greater with the intact antibody than for the fragment, impairing tumour contrast. Fab fragments, approximately 50 kDa in size, have been developed predominantly as imaging agents. A possible disadvantage of Fab fragments is that their molecular weight subjects them to glomerular filtration. High uptake of antibody fragments in the kidneys has been reported before [49,50], most likely a result of reabsorption by the renal tubular cells and catabolism [51]. It is possible that with a larger format, such as Fab_2_ with a size above the renal threshold [52], the fragments would exhibit a biodistribution shift from the kidneys to the liver and may also improve affinity and tumour uptake even further. This will be assessed in a future study.

Another limitation of Fab fragments pertains to the fact that they are monovalent, often resulting in the loss of functional affinity and reduced binding strength compared to e.g. Fab_2_ or IgG. However, *in vitro* evaluations of our Fab fragment using SPR demonstrated *K*_D_ in the low nanomolar range, which is considered optimal for imaging [53]. Studies with ^125^I-labelled Fab fragments with lower affinity (*K*_D_ 30 ± 8 nM) derived from the CD44v6-targeting chimeric antibody U36 [13] have previously been performed in our group. The ^125^I-U36-Fab fragments demonstrated lower tumour uptake and lower tumour-to-blood ratios compared to the ^125^I-Fab in the present study, as well as less optimal tumour-to-organ ratios for e.g. salivary glands, tongue, thyroid and bone. The differences may be the result of many different aspects, for example, tumour cell line and size, as well as administered dose and specific activity of the conjugates. However, since AbD15179 was selected from a large number of fragments due to its high affinity and very slow dissociation from tumour cells [28], one hypothesis for the superior targeting qualities of ^125^I-Fab compared to those of ^125^I-U36-Fab may be the desirable kinetic properties of ^125^I-Fab. Thus, we believe that not only the fully human format but also the suitable affinity of AbD15179 will be an advantage for radio-immunodiagnostics.

## Conclusion

We have, for the first time, evaluated the *in vivo* properties of the AbD15179 Fab fragment targeted against CD44v6 labelled with ^111^In or ^125^I and assessed its utility as a targeting agent for radio-immunotargeting of HNSCC. Our study demonstrated that ^111^In-Fab displayed a superior tumour-to-blood ratio in high-expressing CD44v6 tumours at all time points compared to ^125^I-Fab. Furthermore ^111^In-Fab was able to discriminate between moderate and high CD44v6 expression *in vivo*. We conclude that AbD15179 efficiently targets CD44v6-expressing squamous cell carcinoma xenografts, and particularly, the ^111^In-Fab displayed high and specific tumour uptake. CD44v6 emerges as a suitable target for radio-immunodiagnostics, and a fully human antibody fragment such as AbD15179 can enable further clinical imaging studies.

## Abbreviations

BSA: bovine serum albumin; CAT: chloramine T; CD: cluster of differentiation; CD44v: variable CD44; CD44v6: v6 containing CD44; cMAb: chimeric monoclonal antibody; DMEM: Dulbecco's modified eagle medium; Fc: fragment crystallizables; Fab and Fab_2_: fragment antigen binding; HNSCC: head and neck squamous cell carcinoma; HuCAL: human combinatorial antibody library; IgG: immunoglobulin G; ITLC: instant thin-layer chromatography; *K*_D_: equilibrium dissociation constant; NAP-5: gel filtration column; PBS: phosphate-buffered saline; PET: positron emission tomography; p.i.: post injection; SCC: squamous cell carcinomas; SPECT: single-photon emission tomography; SPR: surface plasmon resonance; U36: monoclonal antibody U36.

## Competing interests

The authors declare that they have no competing interests.

## Authors’ contributions

AKH contributed to the conception and design of the study, carried out the experimental studies and wrote the manuscript. DS, JN, KS and MN contributed to the conception and design of the study, carried out the experimental studies and critically contributed to and revised the manuscript. All authors read and approved the final manuscript.

## Authors’ information

AKH is an otolaryngologist and head and neck surgeon, and a PhD student focusing on methods to detect squamous cell carcinoma in the head and neck region. DS has an MSc degree in Biology and is a PhD student focusing on molecular imaging and therapy of cancer. JN has a PhD degree in biotechnology and is currently a postdoctoral fellow working on antibody engineering. KS is an otolaryngologist and head and neck surgeon with a PhD degree in otorhinolaryngology. MN is an associate professor in Biomedical Radiation Sciences Unit, with a PhD degree in otorhinolaryngology.
